# Update: Product, Substance-Use, and Demographic Characteristics of Hospitalized Patients in a Nationwide Outbreak of E-cigarette, or Vaping, Product Use–Associated Lung Injury — United States, August 2019–January 2020

**DOI:** 10.15585/mmwr.mm6902e2

**Published:** 2020-01-17

**Authors:** Sascha Ellington, Phillip P. Salvatore, Jean Ko, Melissa Danielson, Lindsay Kim, Alissa Cyrus, Megan Wallace, Amy Board, Vikram Krishnasamy, Brian A. King, Dale Rose, Christopher M. Jones, Lori A. Pollack, Lung Injury Response Epidemiology/Surveillance Task Force, Lung Injury Response Epidemiology/Surveillance Task Force, Amena Abbas, Adebola Adebayo, Sukhshant Atti, Elizabeth Carter, Gyan Chandra, Lindsay Eckhaus, Geroncio Fajardo, Sonal Goyal, Benjamin Hallowell, Janet Hamilton, Mia Israel, Zheng Li, Caitlin Loretan, Ruth Lynfield, Paul Melstrom, Mary Pomeroy, Caroline Schrodt, Stephen Soroka, Kimberly Thomas, Bailey M. Wallace

**Affiliations:** ^1^National Center for Chronic Disease Prevention and Health Promotion, CDC; ^2^National Center for Injury Prevention and Control, CDC; ^3^Epidemic Intelligence Service, CDC; ^4^National Center on Birth Defects and Developmental Disabilities, CDC; ^5^National Center for Immunization and Respiratory Diseases, CDC; ^6^Office of Minority Health and Health Equity, CDC; ^7^National Center for Emerging and Zoonotic Infectious Diseases, CDC.; National Center for Chronic Disease Prevention and Health Promotion,; CDC; National Center for Injury Prevention and Control, CDC; Agency For Toxic Substances and Disease Registry; CDC; National Center for Environmental Health, CDC; National Center for Chronic Disease Prevention and Health Promotion, CDC; National Center for Chronic Disease Prevention and Health Promotion, CDC; National Center for Emerging and Zoonotic Infectious Diseases, CDC; National Center for Chronic Disease Prevention and Health Promotion, CDC; National Center for Immunization and Respiratory Diseases, CDC; Council of State and Territorial Epidemiologists; Council of State and Territorial Epidemiologists; Agency For Toxic Substances and Disease Registry, CDC; National Center for Immunization and Respiratory Diseases, CDC; Minnesota Department of Health; National Center for Chronic Disease Prevention and Health Promotion, CDC; National Center for Emerging and Zoonotic Infectious Diseases, CDC; National Center for Emerging and Zoonotic Infectious Diseases, CDC; National Center for Emerging and Zoonotic Infectious Diseases, CDC; Center for Surveillance, Epidemiology, and Laboratory Services, CDC; National Center on Birth Defects and Developmental Disabilities, CDC.

CDC, the Food and Drug Administration (FDA), state and local health departments, and public health and clinical stakeholders continue to investigate a nationwide outbreak of e-cigarette, or vaping, product use–associated lung injury (EVALI) (*1*). EVALI patients in Illinois, Utah, and Wisconsin acquired tetrahydrocannabinol (THC)-containing products primarily from informal sources (*2**,**3*). This report updates demographic characteristics and self-reported sources of THC- and nicotine-containing e-cigarette, or vaping, products derived from EVALI patient data reported to CDC by state health departments. As of January 7, 2020, among 1,979 (76%) patients with available data on substance use, a total of 1,620 (82%) reported using any THC-containing products, including 665 (34%) who reported exclusive THC-containing product use. Use of any nicotine-containing products was reported by 1,128 (57%) patients, including 264 (13%) who reported exclusive nicotine-containing product use. Among 809 (50%) patients reporting data on the source of THC-containing products, 131 (16%) reported acquiring their products from only commercial sources (i.e., recreational dispensaries, medical dispensaries, or both; vape or smoke shops; stores; and pop-up shops), 627 (78%) from only informal sources (i.e., friends, family, in-person or online dealers, or other sources), and 51 (6%) from both types of sources. Among 613 (54%) EVALI patients reporting nicotine-containing product use with available data on product source, 421 (69%) reported acquiring their products from only commercial sources, 103 (17%) from only informal sources, and 89 (15%) from both types of sources. Adolescents aged 13–17 years were more likely to acquire both THC- and nicotine-containing products from informal sources than were persons in older age groups. The high prevalence of acquisition of THC-containing products from informal sources by EVALI patients reinforces CDC’s recommendation to not use e-cigarette, or vaping, products that contain THC, especially those acquired from informal sources. Although acquisition of nicotine-containing products through informal sources was not common overall, it was common among persons aged <18 years. While the investigation continues, CDC recommends that the best way for persons to ensure that they are not at risk is to consider refraining from the use of all e-cigarette, or vaping, products.

This report updates patient demographic characteristics, self-reported substance use, and e-cigarette, or vaping, product sources reported to CDC as of January 7, 2020. States and jurisdictions voluntarily report data on confirmed and probable hospitalized or deceased EVALI patients to CDC weekly using established case definitions* and data collection tools.^†^ Data on substance use and product source were collected from EVALI patients or their proxies (e.g., family members) via standard interview. Commercial product sources were defined as recreational or medical dispensaries, vape or smoke shops, stores, and pop-up shops. Informal sources were defined as friends, family, in-person or online dealers, or other sources. Severe clinical course was defined as hospital stay of ≥10 days; admission to an intensive care unit; requirement for endotracheal intubation, continuous positive airway pressure, or bilevel positive airway pressure; or death. All analyses were conducted using R software (version 3.6; R Foundation for Statistical Computing). The association of age group and product source was tested using Fisher’s exact test, with p-values <0.05 considered statistically significant.

As of January 7, 2020, among 1,979 (76%) patients with substance use data available, 1,620 (82%) reported using any THC-containing e-cigarette, or vaping, products, and 665 (34%) (i.e., 41% of patients reporting any THC-containing product use) reported exclusive use of these products (Table). Among patients reporting any THC-containing product use, 865 (53%) had data on frequency of use; 641 (74%) reported daily use, and 122 (14%) reported using these products a few times per week. Among EVALI patients reporting any THC-containing product use, 809 (50%) reported product source, including 131 (16%) who reported acquiring products from only commercial sources, 627 (78%) from only informal sources, and 51 (6%) from both sources. The most common sources reported for THC-containing products were family members or friends (38%), followed by dealers (31%), and other sources (23%). Medical dispensaries were reported as a source for THC-containing products by 3% of EVALI patients and recreational dispensaries by 8% of EVALI patients.

**TABLE:** Demographic characteristics, substances used, and product sources among hospitalized* cases of e-cigarette, or vaping, product use–associated lung injury (EVALI) reported to CDC — United States, August 2019–January 2020^†^

Characteristic	Substance used No./Total no. (%)				All cases (N = 2,602)
	Any THC (N = 1,620)	Exclusive THC^§ ^(N = 665)	Any nicotine (N = 1,128)	Exclusive nicotine^¶ ^(N = 264)	
**Sex**					
Male	1,116/1,613 (69)	447/662 (68)	759/1,124 (68)	154/264 (58)	1,658/2,486 (67)
Female	497/1,613 (31)	215/662 (32)	365/1,124 (32)	110/264 (42)	828/2,486 (33)
**Age group (yrs)**					
13–17	272/1,615 (17)	93/663 (14)	204/1,125 (18)	32/264 (12)	383/2,497 (15)
18–24	630/1,615 (39)	214/663 (32)	481/1,125 (43)	87/264 (33)	931/2,497 (37)
25–34	387/1,615 (24)	180/663 (27)	239/1,125 (21)	62/264 (23)	605/2,497 (24)
35–44	200/1,615 (12)	98/663 (15)	115/1,125 (10)	35/264 (13)	322/2,497 (13)
45–64	110/1,615 (7)	65/663 (10)	68/1,125 (6)	34/264 (13)	213/2,497 (9)
65–85	16/1,615 (1)	13/663 (2)	18/1,125 (2)	14/264 (5)	43/2,497 (2)
Race/Ethnicity**					
White	969/1,293 (75)	362/503 (72)	744/940 (79)	175/216 (81)	1,333/1,768 (75)
Black	43/1,293 (3)	16/503 (3)	34/940 (4)	11/216 (5)	64/1,768 (4)
Hispanic	219/1,293 (17)	110/503 (22)	104/940 (11)	18/216 (8)	281/1,768 (16)
Other	62/1,293 (5)	15/503 (3)	58/940 (6)	12/216 (6)	90/1,768 (5)
**Clinical course**					
Severe	538/1,600 (34)	211/649 (33)	409/1,122 (36)	106/262 (40)	810/2,533 (32)
Not severe	1,062/1,600 (66)	438/649 (67)	713/1,122 (64)	156/262 (60)	1,723/2,533 (68)
**Outcome**					
Died	28/1,493 (2)	16/597 (3)	26/1,060 (2)	16/244 (7)	57/2,355 (2)
Survived	1,465/1,493 (98)	581/597 (97)	1,034/1,060 (98)	228/244 (93)	2,298/2,355 (98)
**E-cigarette, or vaping, substances reported** ^††^					
Any THC	1,620/1,620 (100)	665/665 (100)	811/1,128 (72)	N/A	1,620/1,979 (82)
Any nicotine	811/1,620 (50)	N/A	1,128/1,128 (100)	264/264 (100)	1,128/1,979 (57)
Any CBD	251/1,620 (15)	N/A	154/1,128 (14)	N/A	308/1,979 (16)
Any other substances^§§^	115/1,620 (7)	N/A	111/1,128 (10)	N/A	158/1,979 (8)
**THC use frequency**					
Daily	641/865 (74)	225/294 (77)	331/468 (71)	N/A	641/865 (74)
A few times per week	122/865 (14)	48/294 (16)	61/468 (13)	N/A	122/865 (14)
A few times per month	49/865 (6)	5/294 (2)	41/468 (9)	N/A	49/865 (6)
Monthly or less	53/865 (6)	16/294 (5)	35/468 (7)	N/A	53/865 (6)
**Nicotine use frequency**					
Daily	407/481 (85)	N/A	580/681 (85)	135/160 (84)	580/681 (85)
A few times per week	39/481 (8)	N/A	55/681 (8)	14/160 (9)	55/681 (8)
A few times per month	17/481 (4)	N/A	22/681 (3)	5/160 (3)	22/681 (3)
Monthly or less	18/481 (4)	N/A	24/681 (4)	6/160 (4)	24/681 (4)
**THC source**					
Pop-up shop^¶¶^	20/783 (3)	9/277 (3)	6/423 (1)	N/A	20/783 (3)
Recreational dispensary^¶¶^	63/783 (8)	26/277 (9)	28/423 (7)	N/A	63/783 (8)
Medical dispensary^¶¶^	27/783 (3)	10/277 (4)	14/423 (3)	N/A	27/783 (3)
Vape or smoke shop^¶¶^	44/783 (6)	15/277 (5)	23/423 (5)	N/A	44/783 (6)
Store^¶¶^	15/783 (2)	4/277 (1)	10/423 (2)	N/A	15/783 (2)
Family or friend***	294/783 (38)	99/277 (36)	174/423 (41)	N/A	294/783 (38)
Dealer***	240/783 (31)	82/277 (30)	140/423 (33)	N/A	240/783 (31)
Online***	43/783 (5)	19/277 (7)	19/423 (4)	N/A	43/783 (5)
Other***	177/783 (23)	62/277 (22)	86/423 (20)	N/A	177/783 (23)
Only commercial sources	131/809 (16)	47/285 (16)	61/436 (14)	N/A	131/809 (16)
Only informal sources	627/809 (78)	216/285 (76)	352/436 (81)	N/A	627/809 (78)
Commercial and informal	51/809 (6)	22/285 (8)	23/436 (5)	N/A	51/809 (6)
**Nicotine source**					
Pop-up Shop^¶¶^	2/430 (0)	N/A	2/595 (0)	0/131 (0)	2/595 (0)
Recreational dispensary^¶¶^	7/430 (2)	N/A	7/595 (1)	0/131 (0)	7/595 (1)
Vape or smoke shop^¶¶^	197/430 (46)	N/A	287/595 (48)	67/131 (51)	287/595 (48)
Store^¶¶^	188/430 (44)	N/A	253/595 (43)	54/131 (41)	253/595 (43)
Family or friend***	76/430 (18)	N/A	91/595 (15)	13/131 (10)	91/595 (15)
Dealer***	15/430 (3)	N/A	15/595 (3)	0/131 (0)	15/595 (3)
Online***	40/430 (9)	N/A	54/595 (9)	10/131 (8)	54/595 (9)
Other***	42/430 (10)	N/A	57/595 (10)	12/131 (9)	57/595 (10)
Only commercial sources	289/442 (65)	N/A	421/613 (69)	105/136 (77)	421/613 (69)
Only informal sources	77/442 (17)	N/A	103/613 (17)	23/136 (17)	103/613 (17)
Commercial and informal	76/442 (17)	N/A	89/613 (15)	8/136 (6)	89/613 (15)

**Abbreviations:** CBD = cannabidiol; N/A = not applicable; THC = tetrahydrocannabinol.

* Includes all hospitalized EVALI patients and EVALI-associated deaths (n = 57) regardless of hospitalization status.

^†^ For cases reported as of January 7, 2020.

^§^ Exclusive THC use defined as anyone who reported THC and no other substances (e.g., nicotine, CBD, synthetics, flavors, or other).

^¶^ Exclusive nicotine use defined as anyone who reported nicotine and no other substances (e.g., THC, CBD, synthetics, flavors, or other).

** Whites, blacks, and others were all non-Hispanic. Hispanic persons could be of any race.

^††^ In the 3 months preceding symptom onset.

^§§^ Includes synthetic cannabinoids and flavors.

^¶¶^ Commercial source.

*** Informal source.

Overall, 1,128 (57%) patients reported using any nicotine-containing products, and 264 (13%) (i.e., 23% of patients reporting any nicotine-containing product use) reported exclusive use of these products. Among 681 (60%) patients with data available on frequency of nicotine-containing product use, 580 (85%) reported daily use, with a similar percentage among exclusive (84%) users. Among EVALI patients reporting use of any nicotine-containing product, 613 (54%) reported product source, including 421 (69%) who reported acquiring products from only commercial sources, 103 (17%) from only informal sources, and 89 (15%) from both sources. Among EVALI patients reporting use of any nicotine-containing products, the most commonly reported sources for nicotine-containing products were vape or smoke shops (48%), stores (43%), and family members or friends (15%).

Younger age was significantly associated with acquiring THC-containing and nicotine-containing products through informal sources (Figure 1). Among EVALI patients reporting use of any THC-containing products, 122 of 130 (94%) of those aged 13–17 years acquired products through only informal sources, compared with 42 of 68 (62%) of those aged 45–77 years (p<0.001). Among EVALI patients reporting use of any nicotine-containing products, 46 of 109 (42%) of those aged 13–17 years acquired products through only informal sources, compared with five of 43 (12%) of those aged 45–75 years (p<0.001).

**FIGURE 1:**
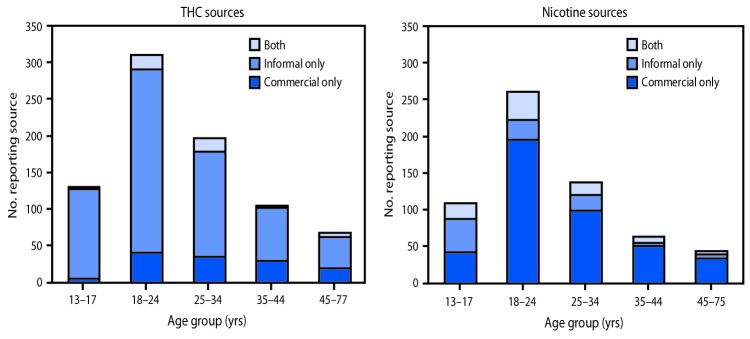
Reported product sources,*^,†^^,^^§^ by age group,^ ¶^^,^** among hospitalized e-cigarette, or vaping, product use–associated lung injury (EVALI) patients — United States, August 2019–January 2020 **Abbreviation:** THC = tetrahydrocannabinol. * Among 809 EVALI patients reporting use of THC-containing products and for whom data on product source (commercial or informal) and age were available. ^†^ Among 613 EVALI patients reporting use of nicotine-containing products and for whom data on product source (commercial or informal) and age were available. ^§^ Informal sources are defined as friends, family, in-person or online dealers, or other sources. ^¶^ P<0.001 for comparison of proportions reporting THC source by age. ** P<0.001 for comparison of proportions reporting nicotine source by age.

The percentage of EVALI patients in each state acquiring THC-containing products from informal sources varied (Figure 2). Alaska, Hawaii, Idaho, Iowa, Mississippi, Montana, Oklahoma, Rhode Island, South Dakota, and Vermont had the highest percentages of patients acquiring THC-containing products from informal sources (50–100%). The percentage of EVALI patients acquiring nicotine-containing products from informal sources also varied by state, with Nevada having the highest percentage (57%).

**FIGURE 2:**
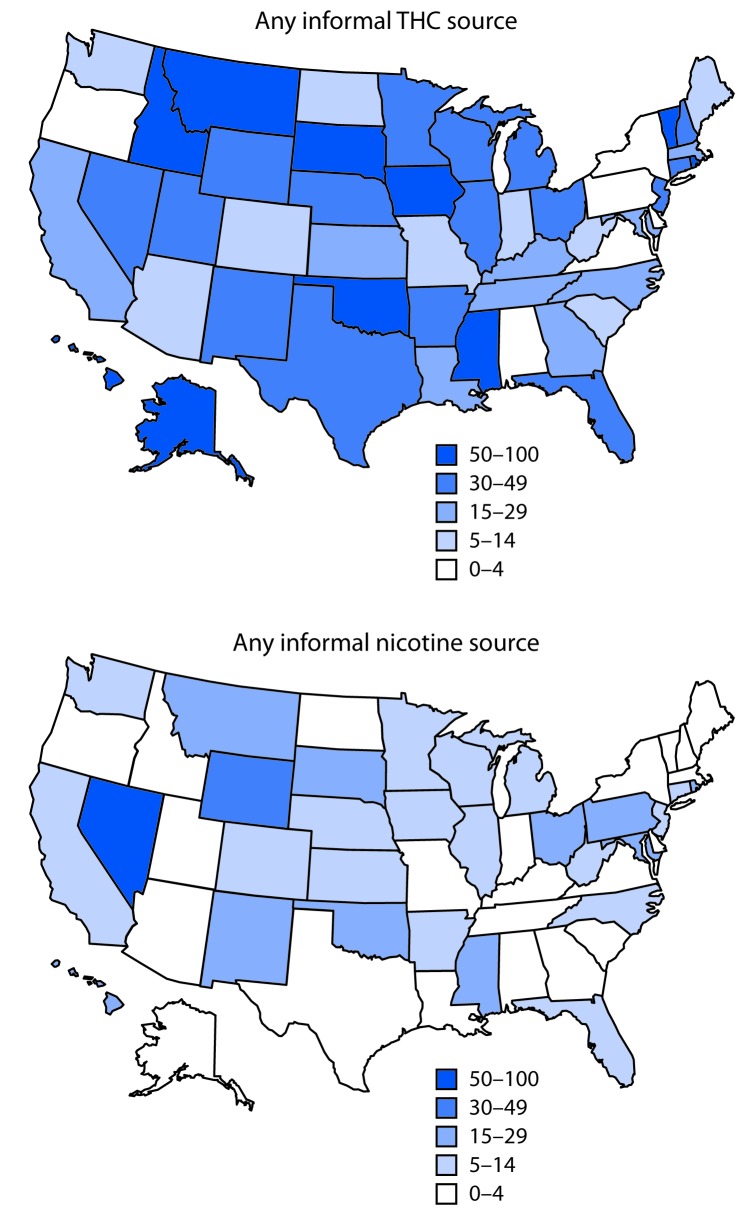
Percentage of hospitalized e-cigarette, or vaping, product use–associated lung injury (EVALI) patients reporting informal product sources,* by state — United States, August 2019–January 2020 **Abbreviation:** THC = tetrahydrocannabinol. * Informal sources are defined as friends, family, in-person or online dealers, or other sources.

## Discussion

Differences in product sources for THC- and nicotine-containing products were identified: obtaining products from only informal sources was substantially more common for THC- than for nicotine-containing products, whereas obtaining products only from commercial sources was much more common for nicotine- than for THC-containing products. These findings are consistent with previous reports on EVALI cases from Illinois, Utah, and Wisconsin, which also found that most THC-containing products were acquired from informal sources, whereas most nicotine-containing products were acquired from commercial sources (*2**,**3*).

The reported use of THC-containing products from informal sources by most EVALI patients is important because vitamin E acetate has been detected in products obtained from these sources and has been associated with EVALI. As part of the investigation into the nationwide outbreak, FDA has conducted testing on products obtained from 73 EVALI patients; 79% of them had at least one product test positive for THC; among those, 78% had at least one product test positive for vitamin E acetate.^§^ A recent case-control study found vitamin E acetate in the bronchoalveolar lavage fluid of 94% of 51 EVALI patients and in none of 99 healthy controls in the comparator group (*4*). In addition, an analysis of THC-containing products seized by law enforcement in Minnesota found no vitamin E acetate in 10 products seized in 2018, and 100% of 20 products seized in 2019 contained vitamin E acetate (*5*).

Although most EVALI cases have been associated with use of informally sourced THC-containing products, 16% of patients reporting use of THC-containing products reported acquiring them only from commercial sources. Even in states where marijuana has been legalized for recreational use by adults,^¶^ it might be difficult to determine whether a source is licensed through the state. For example, in California, the Bureau of Cannabis Control seized nearly 10,000 illegal vape pens from unlicensed retailers during December 10–12, 2019.** The high prevalence of informally sourced THC-containing products among EVALI patients reinforces current recommendations to not use THC-containing e-cigarette, or vaping, products, particularly those acquired from informal sources.

The findings in this report are subject to at least four limitations. First, data on substances used and product sources were reported by patients or their proxies and might be subject to recall or social desirability bias. A recent study found that among 11 EVALI patients who reported no use of THC-containing e-cigarette, or vaping, products, nine had THC or its metabolites detected in bronchoalveolar lavage fluid (*4*). Second, data on e-cigarette, or vaping, product substances used were missing for 24% of patients overall, and product source was missing for 50% of THC-containing product users and 46% of nicotine-containing product users. Therefore, conclusions derived from these data might not be generalizable to all EVALI patients. Third, patients might not know the contents of their e-cigarette, or vaping, products, which might lead to misclassification of substance use. Finally, EVALI is a diagnosis of exclusion with an intentionally sensitive case definition, and it is possible that cases caused by other etiologies could be misattributed to EVALI.

Vitamin E acetate has been identified as an additive in THC-containing e-cigarette, or vaping, products used by EVALI patients, and laboratory studies have demonstrated that it is associated with lung injury^††^ (*4*–*6*). However, additional research is needed because there might be more than one cause of this outbreak, and some patients report using only nicotine-containing products. Therefore, while the investigation continues, CDC recommends that the best way for persons to ensure that they are not at risk is to consider refraining from the use of all e-cigarette, or vaping, products. Adults using e-cigarette, or vaping, products to quit smoking should not return to smoking cigarettes; they should weigh all risks and benefits and consider using FDA-approved cessation medications.^§§^ Adults who continue to use e-cigarette, or vaping, products should carefully monitor themselves for symptoms and see a health care provider immediately if they develop symptoms similar to those reported in this outbreak (*7*). Irrespective of the ongoing investigation, e-cigarette, or vaping, products should never be used by youths, young adults, or pregnant women.

**Summary**

**What is already known about this topic?**

E-cigarette, or vaping, product use–associated lung injury (EVALI) patients in Illinois, Utah, and Wisconsin acquired tetrahydrocannabinol (THC)-containing products primarily from informal sources.

**What is added by this report?**

Nationwide, most EVALI patients with data on product source reported acquiring THC-containing products from only informal sources, whereas most nicotine-containing products were acquired from commercial sources. EVALI patients aged 13–17 years were more likely to acquire both THC- and nicotine-containing products from informal sources than were adults.

**What are the implications for public health practice?**

While the investigation continues, CDC recommends that the best way for persons to ensure that they are not at risk is to consider refraining from the use of all e-cigarette, or vaping, products.

## Footnotes

* https://www.cdc.gov/tobacco/basic_information/e-cigarettes/assets/2019-Lung-Injury-Surveillance-Case-Definition-508.pdf.

† https://www.cdc.gov/tobacco/basic_information/e-cigarettes/severe-lung-disease/healthcare-providers/pdfs/National-Case-Report-Form-v01.pdf.

§
https://www.fda.gov/news-events/public-health-focus/lung-illnesses-associated-use-vaping-products .

¶ https://teens.drugabuse.gov/blog/post/how-legal-marijuana.

** https://bcc.ca.gov/about_us/documents/media_20191213_2.pdf.

†† https://www.fda.gov/news-events/public-health-focus/lung-illnesses-associated-use-vaping-products.

§§ https://www.cdc.gov/tobacco/campaign/tips/quit-smoking/index.html.
